# Silibinin: an old drug for hematological disorders

**DOI:** 10.18632/oncotarget.19153

**Published:** 2017-07-11

**Authors:** Hai Zou, Xing-Xing Zhu, Guo-Bing Zhang, Yuan Ma, Yi Wu, Dong-Sheng Huang

**Affiliations:** ^1^ Department of Cardiology, Zhejiang Provincial People's Hospital, Hangzhou 310000, China; ^2^ Department of Nephrology, Zhejiang Provincial People's Hospital, Hangzhou 310000, China; ^3^ Department of Pharmacy, Zhejiang Provincial People's Hospital, Hangzhou 310000, China; ^4^ Department of Hematology, Zhejiang Provincial People's Hospital, Hangzhou 310000, China; ^5^ Department of Hepatobiliary Surgery, Zhejiang Provincial People's Hospital, Hangzhou 310000, China; ^6^ People’s Hospital of Hangzhou Medical College, Hangzhou 310000, China

**Keywords:** silibinin, β-thalassemia, acute myeloid leukemia, anaplastic large cell lymphoma, multiple myelomas

## Abstract

**Introduction:**

Silibinin (silybin), a non-toxic natural polyphenolic flavonoid, is the principal and the most biologically active component of silymarin. It is efficient in the treatment of acute and chronic liver disorders caused by toxins, drug, alcohol, hepatitis, and gall bladder disorders. Further, in our previous studies, we explored the anti-cancer efficacy in common cancers, such as lung, prostatic, colon, breast, bladder, as well as, hepatocellular carcinoma. Interestingly, silibinin is still not solely limited to the treatment of these diseases. Recent research endeavors suggest that silibinin may function diversely and serve as a novel therapy for hematological disorders.

**Areas covered:**

It discovered several interesting viewpoints in the widely studied mechanisms of silibinin in the hematological disorders.

**Expert commentary:**

In this report, we review the up-to-date findings of more potency roles of silibinin in β-thalassemia (β-TM), acute myeloid leukemia (AML), anaplastic large cell lymphoma (ALCL) and multiple myelomas (MM) therapy and attempt to clarify the mechanisms underlying its effects. There are two viewpoints: First, The functional mechanisms of silibinin in AML cells via regulating cell differentiation to exert anti-cancer effect; Second, combination treatment strategy may be a good choice.

## INTRODUCTION

**Figure 1:**
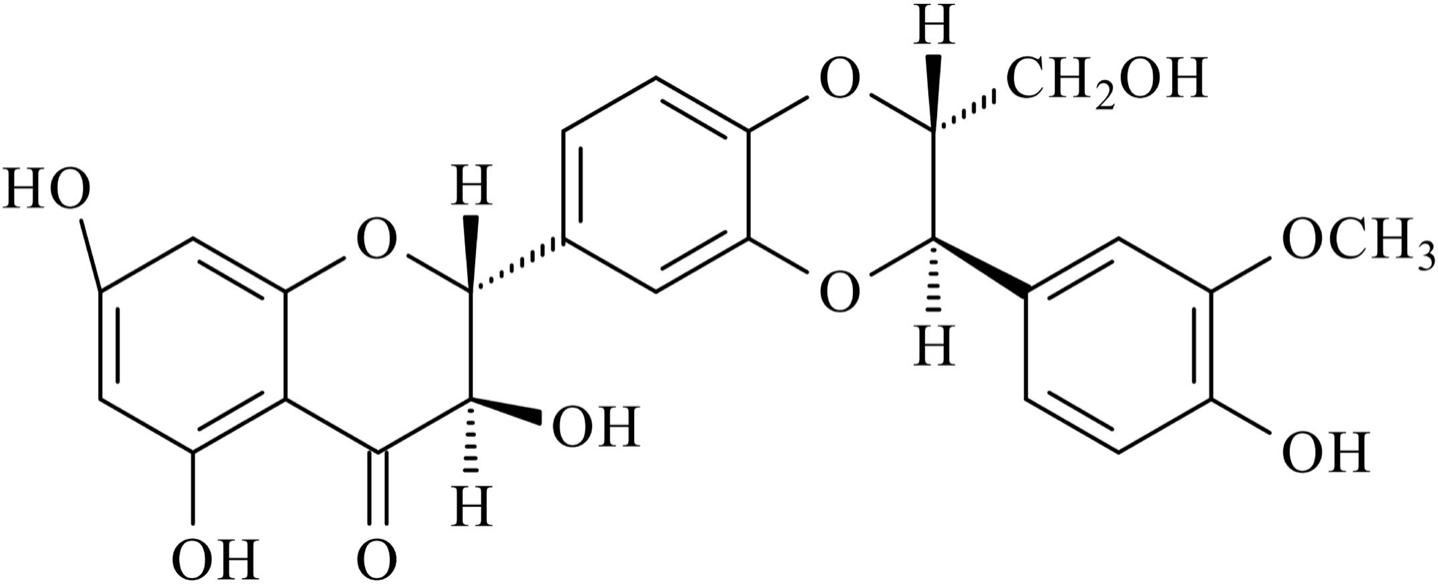
Chemical structures of silibinin

Silibinin (silybin) (Figure 1), is a non-toxic natural polyphenolic flavonoid and the main biologically active component of silymarin. It is efficacious in the treatment of acute and chronic liver disorders caused by toxins, drug, alcohol and hepatitis and gall bladder disorders. Additionally, silibinin has been shown to exert anti-cancer efficacy in well-known cancers, such as lung cancer, prostatic cancer, colon cancer, breast cancer, bladder cancer and also, in hepatocellular carcinoma [1, 2]. In this review we assessed other aspects of silymarin therapeutic potential and summarized the findings regarding the role of the drug in hematological disorders, such as β-thalassemia (β-TM), acute myeloid leukemia (AML), anaplastic large cell lymphoma (ALCL) and multiple myelomas (MM).

### β-thalassemia

β-thalassemia (β-TM) is a chronic hereditary disease, which is characterized by ineffective RBC synthesis due to unbalanced hemoglobin chains production, with the highest prevalence in the Mediterranean Region [3]. Severe chronic anemia, hepatosplenomegaly (SCAH), jaundice, gallstones and bone deformities often develop in homozygous β-TM patients. Although regular blood transfusion could be an effective treatment and reduces mortality, iron overload and oxidative stress have been implicated as the principal precipitating factors of immune deficiency in β-TM major [4, 5]. Furthermore, oxidative stress, inflammation, hepatic involvements, osteoporosis, and cardiac and renal insufficiency are major causes of iron overload related morbidity in patients with β-TM [6]. Studies have demonstrated that reactive oxygen intermediates, such as superoxide anion (O_2_), hydroxyl radicals (OH) and hydrogen peroxide (H_2_O_2_), are in excess in the TM erythrocytes. The reason for this elevation is because of the inhibition of exogenous antioxidant capacity, in particular, vitamin E and zinc inβ-TM [7, 8]. Recently, a series of studies reported that silibinin exerts the property of iron-chelating in patients with β-TM major [9, 10]. For inflammation, a study showed that a significantly higher concentration of TGF-β and IL-23 in the β-TM group than control group, and compared with IL-10 values at baseline, there is a significant reduction in serum IL-10 levels in β-TM patients treated with silibinin. These data suggested that silibinin could be a promising drug for restoring immune response defects in β-TM therapy [11]. A further study indicated that silibinin stimulated a cell-mediated immune response in a β-TM major, possibly through a direct effect on cytokine-producing mononuclear cells, and an indirect action in reducing ferritin and free radical generation during iron-chelating treatment [3]. A recent study showed that silibinin in combination with oral deferasirox (Exjade) could be used safely in the treatment of iron-loaded TM patients as it showed good iron chelation with no sign of toxicity [12]. Moayedi et al. suggested that silibinin was effective at reducing iron overload in patients when used in conjunction with desferrioxamine [10]. It suggests that silibinin possesses the potential effectiveness of silibinin alone in reducing body iron burden [10]. Pieces of evidence have, however, suggested that silibinin could protect β-TM, and considered the likelihood of iron chelation effect with a favorable safety profile. There is the need to conduct more clinical research to evaluate the efficacy of silibinin in β-TM treatment. Hopefully, it will improve the life expectancy of patients with this debilitating illness.

## ACUTE MYELOID LEUKEMIA

Acute myeloid leukemia (AML), a clonal disorder of hematopoietic stem cells, was characterized by an increase in the number of myeloid cells in the marrow and an arrest in their maturation [13]. Dates showed that the incidence of AML ranges from three to five cases per 100,000 population in the United States. Over 10,000 patients died from this disease in 2015 alone [14]. The majority of patients presents with a combination of leukocytosis, anemia, and thrombocytopenia. Subsequently, fatigue, anorexia, and weight loss are common complaints. If patients’ did not accept effective treatment, death usually ensues within months of diagnosis secondary to infection or bleeding [15]. The backbone of therapy of AML has been a combination of cytarabine- and anthracycline-based regimens, and allogeneic stem cell transplantation for eligible candidates [15]. AML is a type of malignancy that is lacking in effective treatment for most patients. The development of new therapies, in concert with proper control of signaling checkpoints in upregulated pathways, as well as, improved genetic profiling, are expected to offer novel and promising methods for AML therapy. Comprehensive studies have demonstrated that silibinin is a novel and promising anticancer agent in AML [16, 17]. Experimental evidence showed that silibinin was capable of markedly suppressing cell growth and produced an anti-proliferative effect, which led to a massive apoptotic cell death in HL-60 and KG-1a human AML cells. Also, time- and dose-dependent cytotoxicity did not substantially accompany these effects [16]. Silibinin exerts an anti-cancer effect based on the by-pass of the block to differentiation caused by the mutations and or epigenetic change in AML cells. What is more, silibinin has been described as phytochemical which could enhance 1,25-dihydroxyvitamin D3 (1,25D), a potent differentiation inducer which has a potential for the treatment of AML- [18] induced differentiation of HL60 myeloblastic leukemia cells [19, 20]. A study suggested that silibinin could induce differentiation as a single agent and increase the levels of several differentiation-related transcription factors (TFs), such as members of jun and CCAAT-enhancer-binding protein (C/EBP) families, which may facilitate association of signal transduction factors during cellular differentiation [21]. Furthermore, silibinin initiated and enhanced 1,25D induced differentiation of AML cells *ex vivo* and induced differentiation more efficiently when cooperated with 1,25D than with non-modified 1,25D [21]. A further study suggested that inhibition of Cot1 by 4-(3-chloro-4-fluorophenylamino)-6-(pyridin-3-yl-methylamino-3-cyano-[1-7]-naphthyridine) or Cot1 siRNA reduced ERK5 activity. This repression will then allow silibinin to increase the differentiation-promoting factors and cell cycle regulators, such as p27/kinase inhibition protein 1 (p27Kip1), which leads to cell cycle arrest, induced by 1,25D in AML cells [22]. Hughes et al. have further shown that silibinin potentiates 1,25D-induced differentiation and growth arrest in AML Cells [18]. This anti-AML effect was related, in part, to upregulation of vitamin D receptor (VDR) and retinoid X receptor (RXRα) levels compatible with increasing transactivation of the vitamin D response element (VDRE).What is more, silibinin activates the Nrf2/Antioxidant Response Element (Nrf2/ARE) signaling pathway, an upstream positive regulator of VDR and RXRα [17]. Cytosine arabinoside (Ara-C) is widely used in the treatment of AML in humans but often becomes ineffective because of increasing resistance to the drug. It is noteworthy that Ara-C in combination with silibinin showed synergistic potential and decreased the IC50 value of Ara-C *in-vitro*. Thus combined treatment is a better strategy with a reduced cytotoxicity profile [23]. However, further study *in vivo* is needed to be carried out to establish the feasibility of this combined therapy in a larger population to firmly determine potency and the underlying mechanism of action. Although silibinin has been demonstrated to exert an anti-cancer effect, more studies are needed in future to establish the relative importance of these signaling molecules in silibinin-induced inhibition of AML cells.

### Anaplastic large cell lymphoma

Anaplastic large cell lymphoma (ALCL) is a distinct subset of T-cell non-Hodgkin lymphomas (NHL). Primary systemic ALCL mostly occurred in childhood, about 40% of NHL cases diagnosed in the pediatric patient are primary systemic ALCL, whereas it just accounts for < 5% of NHL in adults [24, 25]. Nucleophosmin-anaplastic lymphoma kinase (NPM-ALK), an oncogenic fusion protein, has been demonstrated to central the pathogenesis of ALK-positive ALCL (ALK+ALCL) [26]. NPM-ALK is regarded to mediate tumorigenesis via series of cellular pathways, such as phosphatidylinositol 3-kinase (PI3K)/Akt, janus kinase/ signal transducer and activator of transcription 3 (Jak/STAT3), Jun N-terminal kinase (JNK) and MEK/extracellular regulated protein kinase (ERK). All of these pathways can promote cell proliferation, survival, and migration [27–30]. Recently, Molavi et al. have found that the phosphorylation/activation of NPM-ALK and its critical substrates or downstream mediators, such as STAT3, MEK/ERK, and Akt, were efficiently suppressed by silibinin in ALK+ALCL cells. Also, silibinin inhibited the expression of B-cell lymphoma-2 (Bcl-2), survivin and JunB, which were pathogenetic important in ALK+ ALCL and upregulated by NPM-ALK. Silibinin sensitized SRY (sex determining region Y)-box 2 (Sox2), a master transcriptional factor, was shown to be important in maintaining the pluripotency of embryonic stem cells and active ALK+ALCL cells exposed to a widely used chemotherapeutic drug, doxorubicin, in ALCL [31, 32]. Crizotinib, a small molecule competitive inhibitor of anaplastic lymphoma kinase (ALK), has also shown high cytoreductive antitumour activity in anaplastic large-cell lymphoma [33, 34]. Interestingly, it has been observed that, in crizotinib-resistant cells in ALK-rearranged lung cancer, silibinin-induced inhibition of STAT3 worked together with crizotinib to overcome resistance and restore sensitivity [35]. A few studies, however, have reported the anticancer effect of silibinin in ALCL, and thus could be a potential drug for novel ALCL therapy. More research is needed to elucidate further the functional mechanisms of silibinin in ALCL and explore more of its biological prospects for use in treatment.

### Multiple myeloma

Multiple myelomas (MM), a monoclonal tumor of plasma cells, is the second most frequent and age-adjusted hematological malignancy. The incidence of MM is 100,000 per year in the USA and Europe [36]. MM cells were characterized by extensive somatic hypermutation of immunoglobulin genes, high bone marrow dependence, and absence of IgM expression. So far, the treatments of MM are primarily chemotherapies in conjunction with proteasome inhibitors (PIs), bone marrow (BM) transplantation, and antiresorptive agents such as bisphosphonates corticosteroids. Silibinin has, however, been demonstrated to exert anti-multiple myeloma efficacy. Silibinin inhibition of cellular proliferation and increased apoptosis via repression of PI3K/Akt-(mammalian target of rapamycin) mTOR signaling have been recently reported to occur in U266 MM cells [37].

## CONCLUSIONS

**Figure 2:**
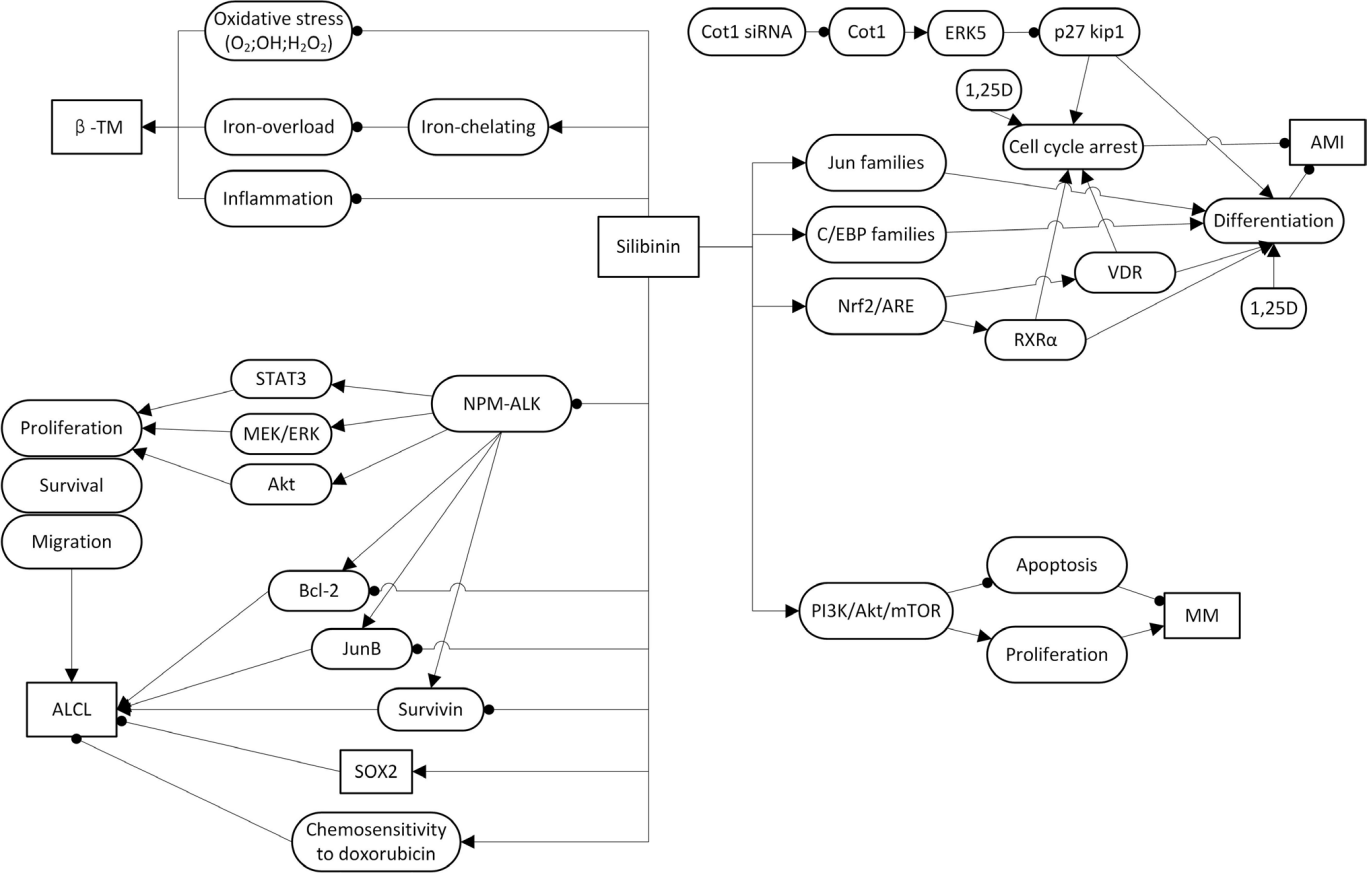
Signaling pathways and the role of silibinin in β-thalassemia; acute myeloid leukemia; Anaplastic large cell lymphoma and multiple myelomas

In this report, we have reviewed the potential of silibinin to potently suppress the hematological disorders, such as β-TM, AML, ALCL, and MM. However, the mechanisms of its effect in displaying these therapeutic properties are not entirely clear; taken together, the evidence from a series of studies showed that silibinin exhibits anti-hematological disorders effects via inhibiting oxidative stress or inducing differentiation and growth arrest in these disease model cell lines (Figure 2). It is well known that iron overload and oxidative stress are the main reasons for immune deficiency in the β-TM major, which was related to the prognosis of β-TM [4, 5]. It is noteworthy that, a series of studies showed that silibinin exerts iron-chelating property and restores immune response defects in patients with β-TM major [9–11]. Further study demonstrated that silibinin function mainly via a direct action on cytokine-producing mononuclear cells and reducing ferritin and free radical generation to mediated immune response in β-TM [3]. As for AML cells, silibinin was capable of markedly suppressing their growth by producing an antiproliferative effect, leading to a massive apoptotic cell death [16]. Further studies have clarified that this anti-AML effect based on the by-pass of the block to differentiation caused by mutations and/or epigenetic change in AML cells. Additionally, silibinin increases the levels of TFs, such as members of jun and C/EBP families [21] and also has been demonstrated to enhance 1,25D differentiation inducing capability potential for differentiation-induced AML treatment [18–21]. Further studies have suggested that inhibition of Cot1 by Cot1 inhibitor, such as Cot1 siRNA, could induce ERK5 activity and subsequently allowed silibinin to increase p27Kip1, which then leads to cell cycle arrest, promoted by 1,25D in AML cells [22]. Nrf2 is persistently activated in AML. Study showed that 1-(4-(tert-Butyl)benzyl)-3-(4-chlorophenyl)-N-hydroxy-1H pyrazole-5-carboxamide (4f), inhibition of Nrf2 , induced apoptosis, at least in part, of human AML cells via Nrf2 signaling [38]. Similarily , it was showed that silibinin might potentiate 1,25D-induced differentiation and growth arrest in AML cells via activation of the Nrf2/ARE signaling pathway, and subsequently upregulate VDR and RXRα levels [17]. A combination of Ara-C and silibinin has been reported to exhibit synergistic potential and decreased the IC50 value of Ara-C *in-vitro*. The underlying mechanism, however, is not well defined [23]. In ALK+ALCL cells, studies showed that silibinin could efficiently suppress the activation of NPM-ALK and its key downstream mediators, such as STAT3, MEK/ERK, and Akt. Besides, it inhibited the expression of Bcl-2, survivin and JunB and upregulation by NPM-ALK. Silibinin could also sensitize Sox2, which is the unique target signaling pathway of silibinin in ALCL, active ALK+ALCL cells to doxorubicin [31, 32]. What’s more, further study showed that Silybum marianum was associated with a trend towards significant reductions in liver toxicity in children with ALL with liver toxicity [39]. In MM cells, silibinin has been demonstrated to be useful in the treatment of MM via inhibition of proliferation and an increase in apoptosis via inhibiting PI3K/Akt-mTOR signaling pathways [37]. These are emerging new ways of silibinin usage which may be promising therapeutic interventions for the treatment of β-TM, AML, ALCL, and MM. There are, however, only a hand full of studies that reflect the use of the drug in an anticancer therapy of ALCL, and MM. Thus more research is needed to enlighten the relevant mechanisms of silibinin action in the treatment of the latter.

## EXPERT OPINION

In this report, we have discussed silibinin in hematological disorders, such as β-TM, AML, ALCL, and MM, considering silibinin as promising therapeutic intervention, based on the studies in the last five years. To this day, a series of studies on the mechanisms of action of silibinin have been carried out, and several experts have highlighted on its potential involvement in the repair of the aberrance in the interaction between signaling pathways, described in (Figure 2 and Table 1) and β-TM, AML, ALCL and MM therapy. We have discovered interesting viewpoints, based on these investigations a) the functional mechanisms of silibinin in AML cells, whether it is through Nrf2/ARE/VDR, Nrf2/ARE/RXRα, jun families or C/EBP families is to eventually regulate cell differentiation to exert an anti-cancer effect [21] [17]. We hypothesized that new signaling pathways by which silibinin regulate cell differentiation in AML cells would be elucidated soon, which will contribute to the effectiveness of silibinin anti-cancer effect to ensure a better understanding; b) a combined treatment strategy may be a good approach. For example, silibinin in combination with 1,25D can obviously potentiate 1,25D-induced differentiation and growth arrest in AML Cells [17]. The combination of Ara-C and silibinin can decrease the IC50 value of Ara-C in AML cells [23]. What’s more, silibinin can increase the chemosensitivity of ALK+ALCL cells to doxorubicin [32]. The inference is that silibinin in combination with doxorubicin would exert a better therapeutic effect than doxorubicin alone. c) It has also been reported that silibinin can inhibit pSTAT3 activation in preclinical cancer models, especially in solid tumors [40]. We also find that, in hematological disorders, silibinin also can suppress the activation of some pathways, such as NPM-ALK, and then suppress its key downstream pSTAT3 [28]. It is noteworthy that, silibinin still have some limitation of clinical use: One of the biggest limitations in humans is its low oral bioavailability [41]. This forces us to seek new formulations. Luckily, recently, interesting clinical antitumor activity has been reported with a new formulation (Eurosil85 - Legasil(R)) in solid cancer patients. We expect that new formulation in this scenario would consider this point to increase oral bioavailability. Although an increasing number of studies explore the role of silibinin in hematological disorders therapy, there are still need extend laboratory studies to help dissect the signaling pathways involved and identify the effect of silibinin in these disorders. For example, in MM, much more cell lines should be presented to represent the whole, genetic, spectrum of patient’s characteristics. In addition, co-cultures with stromal cells may be helpful to mimic the tumour microenvironment and predict effects on drug sensitivity. What’s more, further animal experiments and further clinical studies are need to identify the microenvironment of tumour cells growth, drug resistance, combined drugs, when to be used, primary treatment and the therapeutic effect in relapsed patients of silibinin in hematological disorders therapy.

**Table 1:** Description of different type of hematological disorders

Hematological disorder	Year	Experimental subject	Mechanism of action	Literatures
*β*-TM	2009	human	iron-chelating and reducing iron overload	Gharagozloo et al. [9]
	2013	human	iron-chelating and reducing iron overload	Moayedi et al. [10]
	2013	human	iron-chelating and reducing iron overload	Hagag et al. [12]
	2014	human	inhibiting IL-10 and increasing TGF-b and IL-23	Balouchi et al. [11]
	2013	human	stimulating cell-mediated immune response	Gharagozloo et al. [3]
AML	2010	human AML cells	suppressing cell growth and produced an anti-proliferative effect	Pesakhov et al. [16]
	2001	HL-60 Cells	enhancing protein kinase C (PKC) activity	Kang et al. [19]
	2010	human	increasing jun and C/EBP families	Pesakhov et al. [21]
	2012	HL-60 Cells	activating the Nrf2/ARE)signaling pathway	Wassermann et al. [17]
ALCL	2016	ALK^+^ALCL cells	1.activating of NPM-ALK and suppressing its critical substrates or downstream mediators, such as STAT3, MEK/ERK, and Akt	Molavi et al. [32]
			2. inhibiting the expression of Bcl-2, survivin and JunB	
			3. sensitizing Sox2	
MM	2016	U266 MM cells	inhibiting PI3K/Akt- mTOR signaling	Feng et al. [37]

### Five-year view

Over the next 5 years, we expect the potential effect of therapy of silibinin in hematological disorders. Pharmacological evidence and clinical trial results support the interpretation that silibinin treats hematological disorders by regulating potential signaling pathways, such as Nrf2/ARE/VDR signaling, Nrf2/ARE/RXRα signaling, PI3K/Akt/mTOR signaling and NPM-ALK associated signaling. We predict that extend laboratory studies and further clinical studies will be done to help identify the extend pathogenetic mechanism and the therapeutic effect, usage and safety of silibinin in different hematological disorders. We further predict that silibinin may be approved for the treatment of these hematological disorders in the near future.

### Key issues

• Silibinin, a non-toxic natural compound, may be useful in the treatment of hematological disorders, such as β-TM, AML, ALCL, MM by inducing growth inhibition.

• The treatment strategy of silibinin in combination with some other hemic and lymphatic disease therapeutic drugs for hemic and lymphatic may enhance the therapeutic effects of these drugs

• There are multiple preclinical data supporting activity of silibinin *in vitro* and *in vivo* in patients with β-TM, AML, ALCL, MM, but there are not sufficient published clinical data of efficacious and safety of silibinin treatment in human patients with β-TM, AML, ALCL, MM, especially in ALCL and MM.

## Abbreviations

AKT: protein kinase B(PKB)
ALCL: anaplastic large cell lymphoma
AML: acute myeloid leukemia
BCL-2: B-cell lymphoma-2
C/EBP: CCAAT-enhancer-binding protein
Erk5: extracellular regulated protein kinases 5
MEK/ERK: MEK/extracellular regulated protein kinase
MM: multiple myeloma
Nrf2/ARE: Nrf2/antioxidant response element
NPM-ALK: nucleophosmin-anaplastic lymphoma kinase
p27Kip1: p27/kinase inhibition protein 1
PI3K/Akt/mTOR: phosphatidylinositol 3-kinase/ protein kinase B/ mammalian target of rapamycin
RXRα: retinoid X receptor
STAT3: signal transducer and activator of transcription 3
SOX2: SRY (sex determining region Y)-box 2
VDR: vitamin D receptor
β-TM: β-thalassemia
1,25D: 1,25-dihydroxyvitamin D3
